# Pilot Study on Postoperative Toric Intraocular Lens Alignment: Comparing Casia2 and Photoshop Imaging Techniques

**DOI:** 10.1155/2024/1053914

**Published:** 2024-11-14

**Authors:** Baodi Yang, Chunxin Lai, Yongjie Qin, Hongliang Lin, Sheng Wang, Hailan Liao, Hongyang Zhang

**Affiliations:** ^1^The First School of Clinical Medicine, Southern Medical University, Guangzhou, China; ^2^Department of Ophthalmology, The Second Affiliated Hospital, School of Medicine, Shenzhen & Longgang District People's Hospital of Shenzhen, The Chinese University of Hong Kong, Shenzhen, Guangdong, China; ^3^Department of Ophthalmology, Baiyun Branch, Nanfang Hospital, Southern Medical University, Guangzhou, China; ^4^Department of Ophthalmology, Guangdong Eye Institute, Guangdong Provincial People's Hospital and Guangdong Academy of Medical Sciences, Guangzhou, China; ^5^Department of Ophthalmology, Tohoku University Graduate School of Medicine, Sendai, Japan; ^6^Department of Ophthalmology, West China Hospital, Sichuan University, Chengdu, China; ^7^Department of Ophthalmology, Nanfang Hospital, Southern Medical University, Guangzhou, Guangdong, China

**Keywords:** Adobe Photoshop, alignment, Casia2, digital slit lamp retroillumination, interdevice agreement, pupil diameter, repeatability, toric intraocular lens

## Abstract

**Purpose:** Assess the comparative accuracy and reliability of postoperative toric intraocular lens (TIOL) alignment measurement methods: Casia2 and Adobe Photoshop with digital slit lamp images (PS method).

**Methods:** In a study of 41 subjects with 58 eyes postimplantation of TIOL, we independently measured TIOL alignment with Casia2 and PS methods. The intraclass correlation coefficient (ICC_1,1_) was employed to assess the repeatability of the Casia2 method. While ICC_2,1_ for absolute agreement and Bland–Altman analysis were utilized to determine the interdevice agreement between the two methods, the regression analysis was conducted to identify any proportional bias.

**Results:** Casia2 demonstrated excellent intradevice repeatability with an ICC_1,1_ of 0.998. The absolute agreement between Casia2 and PS was very high with an ICC_2,1_ of 0.999. The average discrepancy between the two measurement methods was −0.828°, with a 95% confidence interval (CI) ranging from −1.623° to −0.032°. The 95% limits of agreement (LoA) were between −6.761° and 5.105°, indicating a strong concordance in TIOL alignment measurements. Casia2 was capable of accurately measuring the TIOL axis alignment under conditions of pupil diameters (PDs) of 4 mm or greater.

**Conclusion:** Casia2 and PS demonstrated significant concordance in measuring postoperative TIOL alignment, with Casia2 offering a more straightforward and accessible alternative, particularly beneficial for patients with suboptimal pupil dilation.

## 1. Introduction

Corneal astigmatism, which affects 34.8% of cataract patients, is a major refractive error [1]. One effective method for reducing preexisting astigmatism is the implantation of toric intraocular lens (TIOL) [2]. However, the toric lens is prone to rotation in the first week after cataract surgery, which can cause a decrease in the correction of astigmatism by 3.3% for every degree of off-axis misalignment [3]. If the misalignment exceeds 10°, surgical repositioning is recommended [4], ideally 2-3 weeks after IOL implantation, prior to the adhesion formation between the TIOL and the capsular bag [5, 6]. Therefore, early detection of IOL rotation is crucial and the method used for measuring postoperative TIOL axial alignment should be accurate and easy to perform.

Currently, the gold standard for evaluating postoperative axial alignment of TIOL is Adobe Photoshop with digital slit lamp images (PS method), which involves taking digital slit lamp retroillumination images combined with Adobe Photoshop software. This method takes into account the tilt of the patient's head and the cyclotorsion of the eye by referring to the episcleral vessel [7, 8]. However, the relative accuracy of the PS method is subject to the observer's experience and bias. In addition, pupils must be sufficiently dilated to visualize one or more toric marks on both sides. With a TIOL optic diameter of 6.0 mm and an overall diameter of 13.0 mm, the minimum pupillary diameter required is 6.5 mm [9].

The Casia2 imaging instrument (Tomey Corporation, Nagoya, Japan) is the second-generation swept-source Fourier-domain anterior segment optical coherence tomography (SS-ASOCT) that has been commercially available since 2015. Compared with the previous generation Casia SS-1000 OCT (Tomey Corporation, Nagoya, Japan), it offers several advantages, including faster scanning speed, higher resolution, and better penetration with a wavelength of 1310 nm, scanning speed of 50,000 A-scans per second, scanning depth of 13 mm, and scanning width of 16 mm. As a result, Casia2 can provide detailed parameters of the classic cornea, anterior chamber, pupil, and lens [10, 11]. In addition, Casia2 has been demonstrated good repeatability and reproducibility under both nonmydriatic and mydriatic conditions [12, 13]. Previous studies had demonstrated the excellent accuracy and simplicity of Casia2 for measuring the tilt and decentration of crystalline lens and IOL automatically [14, 15]. Moreover, the device has built-in software that allows for the measurement of the TIOL axis. However, to our knowledge, there are no reports on the application of Casia2 for measuring the TIOL axis to date.

Therefore, our study aims to introduce the Casia2 method for measuring the alignment of postoperative TIOL and assess the interdevice agreement of the TIOL alignment obtained from Casia2 and PS methods.

## 2. Materials and Methods

### 2.1. Subjects

This retrospective study was performed at the Department of Ophthalmology at Guangdong Provincial People's Hospital. All subjects received toric IOL implantation from January 2021 to December 2022, with models including the AcrySof IQ toric SN6AT IOL (Alcon) and the AT TORBI 709M IOL or 909M multifocal IOL (Carl Zeiss Meditec AG). The TORIC IOLs had an optic diameter of 6.0 mm for an overall length of 13 mm. Patients were excluded from the study if pupils could not be sufficiently dilated to visualize toric marks on both sides, or eyes could not focus on the fixation target during the measurement resulting in unsuccessful detection of the alignment of TIOL. This study was approved by the Ethics Committee of Guangdong Provincial People's Hospital and adhered to the tenets of the Declaration of Helsinki. Written informed consent was obtained from each subject before enrollment in this study.

Prior to cataract surgery, biometric measurements such as corneal curvature, corneal astigmatism, axial length, anterior chamber depth, and lens thickness were obtained using IOL Master 700 (Carl Zeiss Meditec, Germany). All postoperative subjects were scanned with both Casia2 and retroillumination slit lamp photography. Following the administration of mydriatic eye drops, the TIOL axis was first assessed using CASIA2 and subsequently using PS once the pupil dilated sufficiently. The measurements were conducted according to standardized specifications to ensure accuracy and reliability, with subjects positioned in a straight and upright position on the chin rest of the Casia2 or slit lamp devices. All measurements were performed by a single experienced examiner to reduce variability. To assess measurement repeatability, each subject underwent three repeated measurements in a dimly lit room.

### 2.2. Casia2 Method

**Figure 1:**
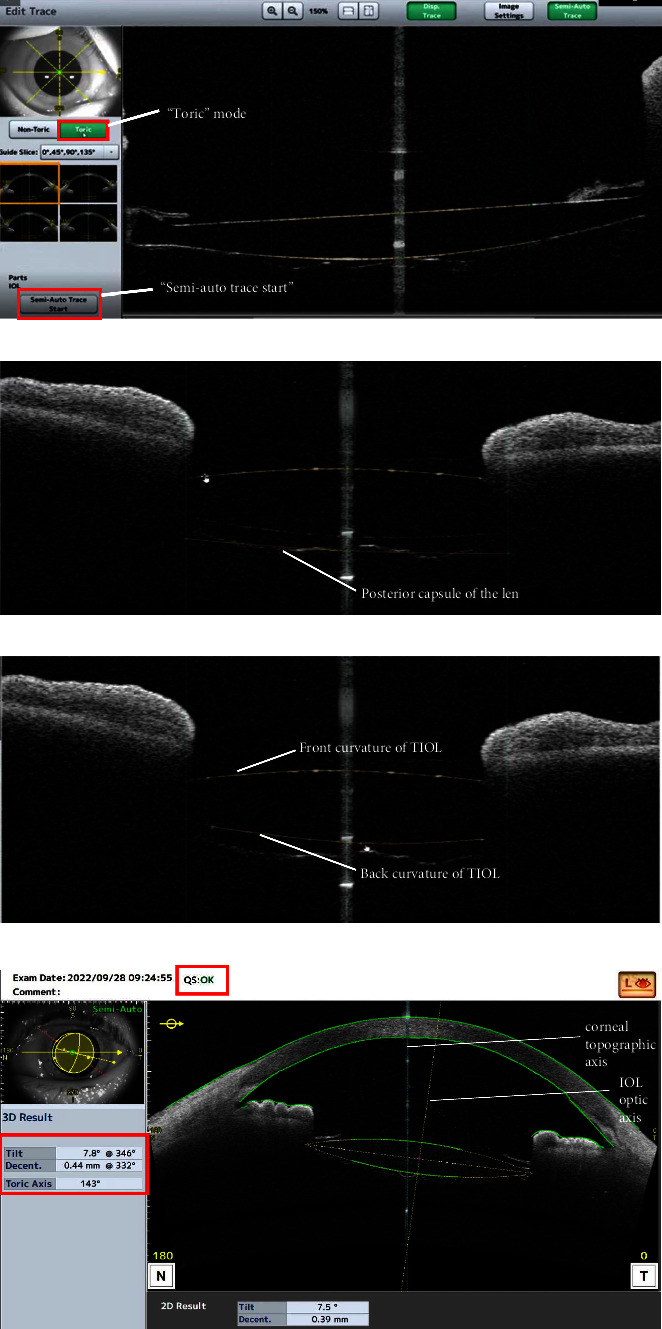
Casia2 method for the measurement of TIOL alignment. TIOL was measured using the IOL scan mode by which the outlines of TIOL were automatically recognized. Then, selected “Toric” mode (a), checked and adjusted the IOL outlines to ensure their accuracy (b, c). Afterward, pressed “Semi-Auto Trace Start” and “Save”, 3D analyses of tilt, decentration and toric axis were generated directly relative to the corneal topographic axis (d).

TIOL were measured using the IOL scan mode, which captures eight distinct AS-OCT images from eight different angles (0–180, 90–270, 23–203, 113–293, 45–225, 135–315, 68–248, and 158–338). The outlines of the TIOL were automatically recognized and then were checked for accuracy by the same examiner, who selected the “Toric” mode and adjusted the IOL outlines as needed. Subsequently, the examiner pressed “Semi-Auto Trace Start” and then “Save” to generate 3D analyses of tilt, decentration, and toric axis relative to the corneal topographic axis (see Figure 1). To ensure consistency, each subject was measured three times by the same examiner using the Casia2 method. And the TIOL axis position was obtained by averaging the three repeated measurements.

### 2.3. PS Method

**Figure 2:**
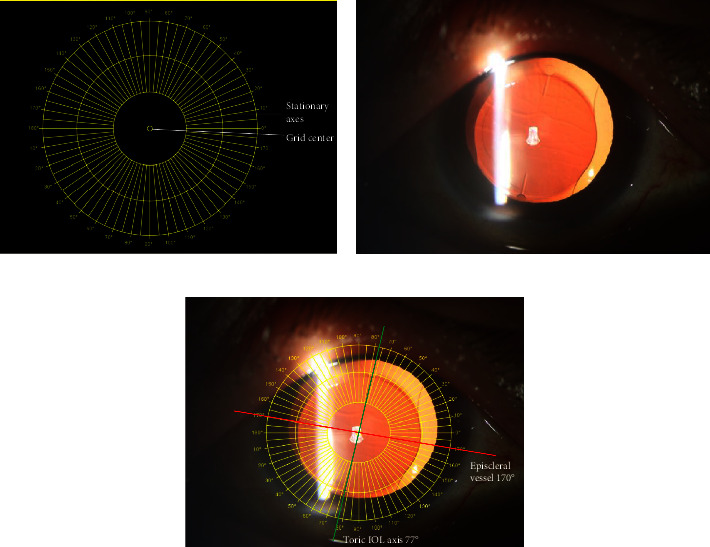
PS method for the measurement of TIOL alignment. (a) The grid ring for the measurement of the TIOL axis can be zoomed in or out by rolling the mouse to adapt to the size of the measured objects; (b) the representative image of digital slit lamp retroillumination with the episcleral vessels and toric axis marks visualized (AT TORBI 709M IOL, Carl Zeiss); (c) overlapped the geometric center of the grid ring and TIOL. The measurement was performed in triplicate to ensure that the orientation (in degrees) and distance of the major episcleral vessel from the geometric center of TIOL were consistent; TIOL axis positions were obtained by averaging the three repeated measurements.

The PS method for measuring the TIOL alignment was based on an objective image analysis technique described by Shah [8]. In brief, high-resolution, slit lamp digital retroillumination photographs were captured with a sufficiently dilated pupil, which is 6.5 mm or greater. A grid ring was used to measure the TIOL axis, which could be zoomed in or out by rolling the mouse to adapt to the size of the measured objects. The geometric center of the grid ring was overlapped onto that of TIOL, and the measurement was performed in triplicate to ensure that the orientation (in degrees) and distance of the major episcleral vessel from the geometric center of the TIOL were consistent. TIOL axis positions were obtained by averaging the three repeated measurements, with deviation caused by head tilt and eyeball cyclotorsion minimized by referencing episcleral vessels (Figure 2). These measures were performed by an experienced examiner to ensure consistency and accuracy.

### 2.4. Statistical Analysis

Statistical analysis was performed using SPSS (version 26) and GraphPad Prism (version 9.0). Continuous variables were summarized with descriptive statistics (mean, standard deviation, median, and range), and categorical variables were expressed as counts and percentages. The intraclass correlation coefficient (ICC_1,1_) was employed to assess the repeatability of the Casia2 method [16, 17]. The interdevice agreement between Casia2 and PS methods was assessed with ICC_2,1_ for absolute agreement [17] and Bland–Altman analysis [18, 19]. ICCs were interpreted as poor (< 0.75), moderate (0.75 to < 0.9), and good (≥ 0.9) [17]. The 95% limits of agreement (LoA) were calculated from the mean difference ± 1.96 × SD according to the Bland–Altman method, with the Bland–Altman plot visually illustrating the agreement between the two methods [20]. Univariate and multivariate regression analyses were employed to identify the related factors for proportional bias, excluding sphere power and cylinder power of TIOL to avoid multicollinearity in the multivariate regression analysis. A *p* value < 0.05 was considered statistically significant.

## 3. Results

### 3.1. Demographic and Biometric Characteristics

Fifty-eight eyes of 41 subjects (20 males and 21 females) who had undergone toric IOL implantation were included in this study. The mean age of the subjects was 67.03 years (ranging from 12 to 88 years).  Table 1 summarizes the preoperative biometry of the study population, including average keratometry (SE), corneal astigmatism (△*K*), axial length (AL), and anterior chamber depth (ACD), all which were measured using the IOL master700 (Carl Zeiss Meditec, Germany).

Bilateral eyes were included in 17 patients. To show that the TIOL axis of both eyes were intraindividually independent, we calculated the ICC_2,1_ between both eyes in the same patient for the measured method of PS and Caisa2 separately, showing no significant correlation between both eyes of the same patient, no matter by PS or Casia2 method (ICC_2,1 casia2_ = 0.094, *P*_Casia2_=0.338; ICC_2,1 PS_ = 0.087, *P*_PS_=0.349).

### 3.2. TIOL Parameter Measurements and Intradevice Agreement

Table 2 shows the implanted TIOL parameters, including the models, sphere power, and cylinder power of the TIOL. Tilt, decentration, and pupil diameter (PD) were measured using Casia2 device, and the PD was that of the Casia2 method in the measurement of the TIOL axis. Casia2 demonstrated excellent intradevice repeatability with an ICC_1,1_ of 0.998 (range from 0.997 to 0.999).

### 3.3. Interdevice Agreement and Bland–Altman Analysis

**Figure 3:**
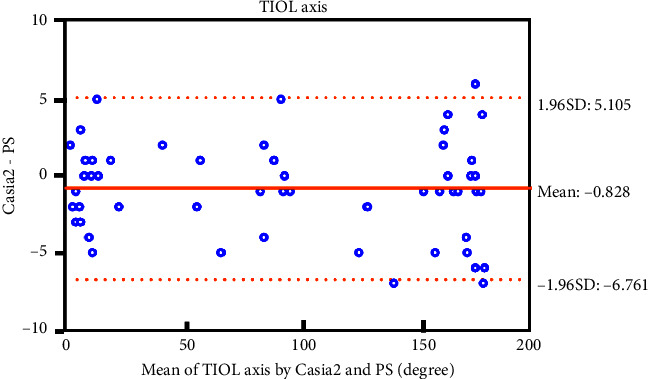
Bland–Altman plot illustrated a strong concordance between Casia2 and PS for the measurement of TIOL axis. The solid orange line indicates the mean difference (bias = −0.828), and the dotted orange lines indicate the 95% limit of agreement (LoA).

The mean value ± standard deviation of the TIOL axis measured was 94.530 ± 69.838° by the Casia2 method and 95.360° ± 70.105° by the PS method, separately. The ICC_2,1_ for absolute agreement was 0.999 (ranging from 0.998 to 0.999), indicating good agreement between the two methods. The mean difference between the two methods (namely, Casia2 and PS) was −0.828°, with a 95% confidence interval (CI) ranging from −1.623° to −0.032°. The 95% LoA were between −6.761° and 5.105°. The Bland–Altman plot (Figure 3) visually illustrated a strong concordance between the two methods.

### 3.4. Agreement Under Different PDs

Table 3 presents the ICC_2,1_ values for assessing the agreement of TIOL axis measurements between Casia2 and PS methods under different PDs. The results showed that Casia2 was capable of accurately measuring the TIOL axis alignment under conditions of PDs of 4 mm or greater.

### 3.5. Univariate and Multivariate Regression Analyses

Table 4 shows the results of univariate and multivariate regression analyses for the absolute difference (AD) as the dependent variable. The results indicated that sex, age, AL, ACD, SE, △*K*, models of IOL, PD, tilt, and decentration were not significantly associated with the AD. Together, the Casia2 device showed excellent intradevice repeatability and interdevice agreement with the PS method in measuring the TIOL axis. The Casia2 method was accurate for the measurement of the TIOL axis when the PD was 4 mm or greater. No significant factors were found to be associated with AD.

## 4. Discussion

As patients increasingly expect optimal postoperative refractive outcomes with minimal astigmatism after cataract surgery, the application of TIOL is more and more common. Measurement of the postoperative TIOL alignment is essential to evaluate the rotational stability of TIOL and guide the repositioning to achieve satisfactory postoperative outcomes.

For the first time, our study introduced the Casia2 method applied for the measurement of the postoperative TIOL axis and evaluated the agreement of measured values between Casia2 and PS for the postoperative TIOL alignment.

In our study, Casia2 showed good reproducibility (ICC_1,1_ 0.998). Previous literature studies also reported good reproducibility with Casia2 in measuring anterior segment parameters (ICC 0.86–0.99) [21, 22]. The reproducibility in our study was superior to those reported in the literature studies that were probably attributed to our operation performed by the same trained operator following a standardized procedure and the result only for the postoperative TIOL axis.

Our study demonstrated that Casia2 and PS had good agreement for the measurement of postoperative TIOL alignment with the ICC analysis (ICC_2,1_ 0.999) and Bland–Altman plot (mean difference −0.828°). Although the 95% LoA seem wide (−6.761 to 5.105°), rotations less than 10° generally result in less than 0.50D of astigmatism, which may be no clinically significant [23]. Therefore, the measured discrepancy from the two methods could be within permissible limits.

In terms of operation, one advantage of Casia2 over PS is its simplicity and user-friendliness for both clinicians and patients. PS requires subjects' pupils to be dilated for at least 6.5 mm so that the toric marks can be seen. While our study demonstrated that, as long as sufficient light entered the eyes—typically with a PD of 4 mm or greater—Casia2 could obtain accurate measurements of the TIOL axis. And the result is similar with previous literature reports that Casia2 has good repeatability and reproducibility under both nonmydriatic and mydriatic conditions [13, 14]. This makes Casia2 a more accessible tool, especially for patients with contraindicated or insufficient mydriasis. In addition, the Casia2 device has an internal fixating target to better control the subject's eye position, reducing the error or bias induced by eyeball cyclotorsion or tilt.

In the past few decades, assessments of tilt and decentration of the IOL had been conducted using Purkinje imaging technique and Scheimpflug imaging. However, they require image-processing software to calculate IOL tilt and decentration, and good pupil dilation is a prerequisite for them to capture high quality images. In addition, they used the pupillary axis as a reference for calculating the tilt and decentration [24–26]. Anterior segment ocular coherence tomography (AS-OCT) is later employed for measurement of crystalline lens and IOL tilt and decentration. Compared with the first generation AS-OCT of Casia SS-1000, Casia2, the second generation of AS-OCT improves the scanning speed from 3000 A scan per second to 5000 A scan per second and increases the scanning depth from 6 to 13 mm, and thus, Casia2 can obtain the image from the anterior corneal surface to the posterior surface of the lens in one shot [14, 27, 28]. In our study, we employed the device of Casia2 for measuring IOL tilt and decentration. Owing to its good penetration, it can automatically and quantitatively measure the tilt and decentration of crystalline lens and IOL with high repeatability, regardless of whether the eye is under mydriasis or not. And it measures the tilt and decentration relative to the corneal topographic axis. Compared with the pupillary axis, the corneal topographic axis is considered a better reference for assessing IOL tilt and decentration because it is not affected by the shape of the pupil [14, 28, 29].

However, our study has several limitations. Firstly, the study was performed with a small sample size and at a single institution, and the number of different models of TIOL was unbalanced, which could limit the generalizability of the study and may even bias its results. Therefore, further studies with large-scale, multicenter, and more balanced TIOL models are required to validate our findings in this study. Secondly, our study is limited to the evaluation of agreement between the two methods. Further studies including refractive outcomes are desirable to determine the precision and accuracy of the two different methods.

## 5. Conclusions

In conclusion, our study demonstrates that Casia2 and PS have significant concordance in measuring the postoperative TIOL alignment. The simplicity and user-friendliness of Casia2 make it a more accessible alternative, particularly beneficial for patients with suboptimal pupil dilation.

## Figures and Tables

**Table 1:** Participant characteristics.

Characteristics	Value
Subjects, (*n*)	41
Eyes, *n* (right/left)	58 (24/34)
Sex (male/female)	20/21
Age, (y)	
Mean ± SD	67.03 ± 20.6
Median (range)	75 (12, 88)
SE (D)	
Mean ± SD	43.56 ± 1.58
Median (range)	43.8 (40.32, 46.90)
△*K* (D)	
Mean ± SD	2.18 ± 0.74
Median (range)	2.06 (1.10, 5.03)
AL (mm)	
Mean ± SD	24.91 ± 2.12
Median (range)	24.17 (21.22, 30.85)
ACD (mm)	
Mean ± SD	2.98 ± 0.48
Median (range)	2.88 (1.99, 3.85)

*Note:* △*K*: Corneal astigmatism; preoperative biometry was performed by IOL master 700 (Carl Zeiss Meditec, Germany).

Abbreviations: ACD, anterior chamber depth; AL, axis length; SE, average keratometry.

**Table 2:** Toric IOL parameters.

Parameters	Value
Models	
Zeiss 709, *n* (%)	40 (69.0%)
Zeiss 909, *n* (%)	9 (15.5%)
Alcon, *n* (%)	9 (15.5%)
Sphere power (D)	
Mean ± SD	16.32 ± 5.31
Median (range)	18.25 (5, 26.5)
Cylinder power (D)	
Mean ± SD	3.03 ± 0.84
Median (range)	3.0 (1.5, 6)
Tilt degree (°)	
Mean ± SD	4.92 ± 1.84
Median (range)	4.90 (1.10, 8.70)
Decentration (mm)	
Mean ± SD	0.22 ± 0.15
Median (range)	0.19 (0.01, 0.70)
PD (mm)	
Mean ± SD	5.69 ± 1.19
Median (range)	5.79 (2.61, 8.80)

*Note:* PD: pupil diameter of Casia2 method for the measurement of TIOL axis. PD, tilt and decentration were measured by Casia2 (Tomey, Japan).

**Table 3:** The ICCs for TIOL axis measurements between Casia2 and PS under different PDs.

PD (mm)	Case (*n*, %)	Casia2 vs. PS	
		ICC_2,1_	95% CI of ICC
< 4	3 (5%)	0.227	−0.280, 0.955
4∼5	14 (24%)	0.999	0.998, 1
5∼6	17 (29%)	0.999	0.997, 1
6∼7	17 (29%)	0.999	0.997, 1
> 7	7 (12%)	1	0.998, 1

*Note:* PD: Pupil diameter of the Casia2 method for the measurement of the TIOL axis.

Abbreviations: CI, confidence interval; ICC, intraclass correlation coefficient.

**Table 4:** Univariate and multivariate regression analyses of the associations between the AD and the relevant parameters.

Variables	Univariate analysis			Multivariate analysis	
	Regression coefficient (95% CI)	*R* ^2^	*p*	Regression coefficient (95% CI)	*p*
Sex	−0.098 (−1.150, 0.955)	0.001	0.853	−0.143 (−1.398, 1.112)	0.819
Age (y)	0.018 (−0.008, 0.043)	0.034	0.167	0.004 (−0.039, 0.048)	0.843
AL (mm)	−0.153 (−0.400, 0.094)	0.027	0.220	−0.288 (−0.759, 0.184)	0.226
ACD (mm)	−0.529 (−1.633, 0.574)	0.016	0.341	1.200 (−1.024, 3.424)	0.283
SE (D)	0.023 (−0.312, 0.359)	0	0.889	−0.029 (−0.461, 0.403)	0.893
△*K* (D)	−0.406 (−1.120, 0.307)	0.023	0.259	−0.823 (−1.907, 0.262)	0.134
Models of IOL	−0.252 (−0.952, 0.448)	0.009	0.474	−0.213 (−1.062, 0.635)	0.615
PD (mm)	−0.239 (−0.681, 0.204)	0.020	0.284	−0.130 (−0.635, 0.375)	0.606
Tilt (degree)	0.100 (−0.188, 0.388)	0.009	0.488	0.217 (−0.168, 0.602)	0.262
Decentration (mm)	−0.895 (−4.500, 2.711)	0.004	0.621	−0.444 (−4.855, 3.966)	0.840

*Note:* AD: the absolute difference of the TIOL axis measured by Casia2 and PS, (|Casia2 − PS|).

## Data Availability

The data used to support the findings of this study are available from the corresponding author upon request.

## Nomenclature

TIOL: Toric intraocular lens
PS method: Digital slit lamp retroillumination images combined with Adobe Photoshop methods
ICC: Intraclass correlation coefficients
LoA: Limits of agreement
SS-ASOCT: Swept-source Fourier-domain anterior segment optical coherence tomography
AD: Absolute difference of the TIOL axis measured by Casia2 and PS
PD: Pupil diameter of Casia2 method in the measurement of TIOL axis
SE: Average keratometry
△*K*: Corneal astigmatism
AL: Axis length
ACD: Anterior chamber depth
